# Regulation of autophagy by natural polyphenols in the treatment of diabetic kidney disease: therapeutic potential and mechanism

**DOI:** 10.3389/fendo.2023.1142276

**Published:** 2023-08-10

**Authors:** Tongtong Liu, Qi Jin, Liping Yang, Huimin Mao, Fang Ma, Yuyang Wang, Ping Li, Yongli Zhan

**Affiliations:** ^1^ Guang’anmen Hospital, China Academy of Chinese Medical Sciences, Beijing, China; ^2^ China-Japan Friendship Hospital, Institute of Medical Science, Beijing, China

**Keywords:** natural polyphenols, autophagy, diabetic kidney disease, transcription factor EB, lysosome

## Abstract

Diabetic kidney disease (DKD) is a major microvascular complication of diabetes and a leading cause of end-stage renal disease worldwide. Autophagy plays an important role in maintaining cellular homeostasis in renal physiology. In DKD, the accumulation of advanced glycation end products induces decreased renal autophagy-related protein expression and transcription factor EB (TFEB) nuclear transfer, leading to impaired autophagy and lysosomal function and blockage of autophagic flux. This accelerates renal resident cell injury and apoptosis, mediates macrophage infiltration and phenotypic changes, ultimately leading to aggravated proteinuria and fibrosis in DKD. Natural polyphenols show promise in treating DKD by regulating autophagy and promoting nuclear transfer of TFEB and lysosomal repair. This review summarizes the characteristics of autophagy in DKD, and the potential application and mechanisms of some known natural polyphenols as autophagy regulators in DKD, with the goal of contributing to a deeper understanding of natural polyphenol mechanisms in the treatment of DKD and promoting the development of their applications. Finally, we point out the limitations of polyphenols in current DKD research and provide an outlook for their future research.

## 1 Introduction

Diabetic kidney disease (DKD) is a major microvascular complication of diabetes mellitus. As the incidence of diabetes continues to rise, DKD has become one of the fastest-growing causes of chronic kidney disease and its associated morbidity and mortality (1, 2). Simultaneously, the occurrence and severity of kidney disease increase the risk of adverse health outcomes, including cardiovascular disease and cancer, and premature mortality in patients with diabetes (3, 4). There is, therefore, an urgent need to improve the diagnosis and management of DKD. DKD pathogenesis is complex, with various metabolic and hemodynamic alterations involved in the development and progression of DKD (5).

Autophagy protects against DKD development by regulating cellular metabolism and organelle homeostasis, as well as degrading and recycling damaged proteins, macromolecules, and organelles (6). Furthermore, under diabetic conditions, autophagy interacts with multiple intracellular stress signals to maintain cellular integrity and contributes to the clearance of damaged proteins and organelles (7). Conditional knockout mice of autophagy-related genes (Atg5- or Atg7-KO mice) in renal resident cells further demonstrate the essential role of basal autophagy in cellular homeostasis (8–10). However, autophagy is suppressed in DKD, and dysregulation of autophagy and lysosomal homeostasis, therefore, aggravates podocyte injury, glomerulosclerosis, and fibrosis in DKD (11, 12). Autophagy defects in DKD are thought to be caused primarily by advanced glycation end products (AGEs). AGEs are a complex and heterogeneous group of compounds that originate as heterogeneous molecules from the nonenzymatic products of glucose reactions or other saccharide derivatives with proteins or lipids (13). Their presence in cells and tissues can be detected by several methods including competitive immunoassay, skin autofluorescence, and stable isotopic dilution analysis liquid chromatography and tandem mass spectrometry (14). AGEs are endocytosed by renal proximal tubules and degraded by lysosomes, but they can also form inside different renal cell types. High glucose (HG), AGE-rich diet, and decreased renal clearance all have the potential to accelerate AGE formation and accumulation in DKD (15). This may lead to apoptosis and inflammation and thereby lead to DKD progression (16). Accumulating evidence supports the causative role of AGEs in autophagy defects in DKD (17). Conversely, autophagy is thought to play a protective role against AGEs-induced apoptosis. The p62-dependent autophagy, for example, was shown to facilitate the removal of AGEs, and the absence of p62 accelerated the accumulation of AGEs in the soluble and insoluble fractions (18).

Although great efforts have been made to develop effective therapies for DKD, delaying its progression to end-stage renal disease (ESRD) remains a great challenge. Natural polyphenols, which are abundant in fruits, vegetables, spices, and herbs, are known for their health benefits on DKD by improving detachment and apoptosis of podocytes, tubulointerstitial fibrosis, proliferation, excessive matrix production of mesangial cells, and infiltration and phenotypic changes of macrophages, potentially by improving autophagy and lysosomal function (19).

In this review, we critically evaluate the strengths and limitations of natural polyphenols, focusing on their regulation of autophagy to provide a clinical reference for the treatment of DKD.

## 2 Dysregulation of autophagy in DKD

**Figure 1:**
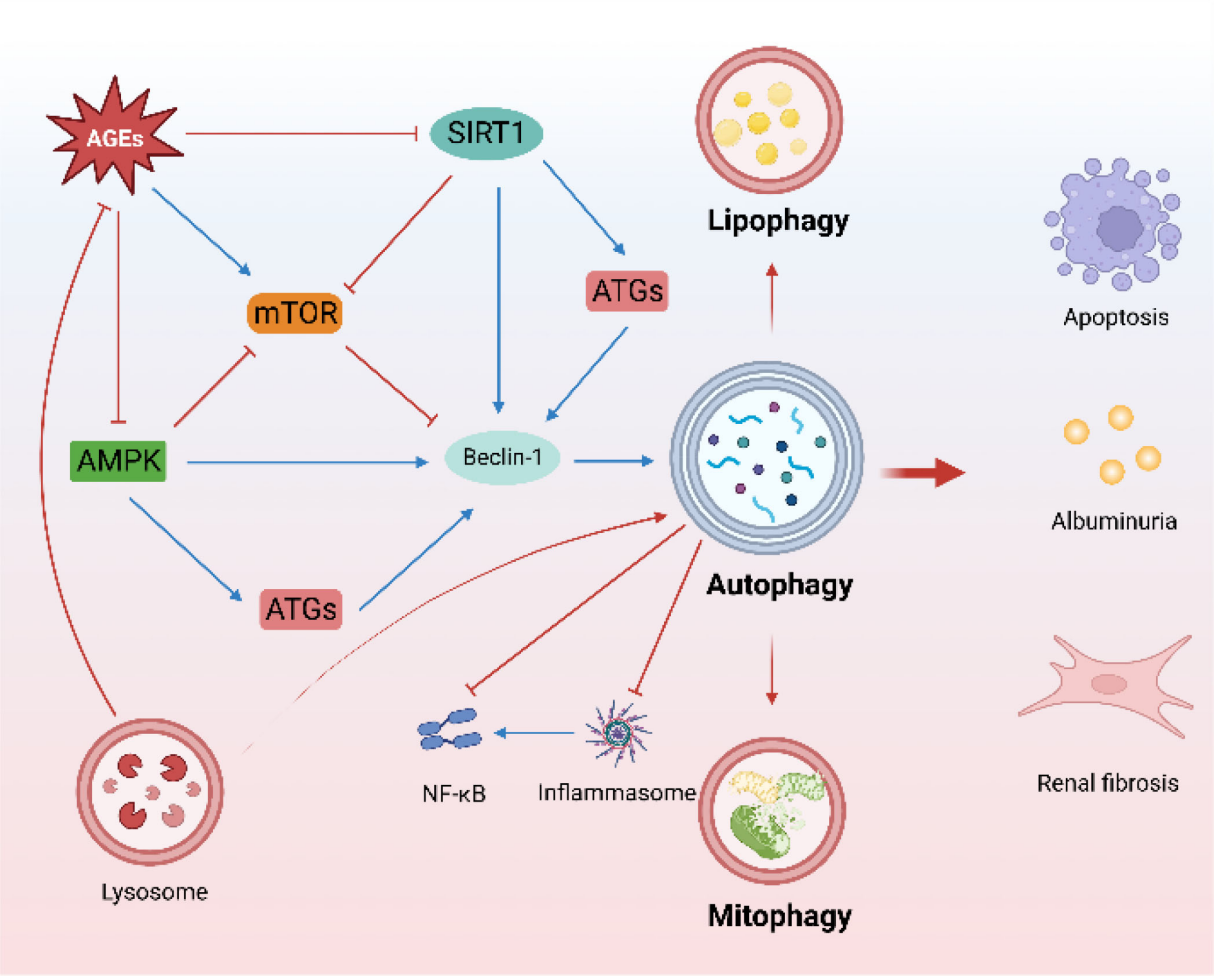
Characteristics of autophagy in DKD. (The mTOR, AMPK, and SIRT1-regulated autophagy pathway is an important protective mechanism for DKD. Mitophagy and lipophagy are critical to DKD. There is a close crosstalk between autophagy and inflammation. Lysosomes promote the degradation of AGEs).

Autophagy and lysosomal dysfunction of renal resident cells are important pathological factors affecting DKD progression. In this section, we discuss the characteristics and regulatory role of autophagy in the onset and progression of DKD and highlight the importance of autophagy regulation in renal resident cells and renal macrophages (**Figure 1**).

### 2.1 Autophagy regulatory pathways

Mammalian target of rapamycin (mTOR), AMP-activated protein kinase (AMPK), and Sirtuin 1 (SIRT1) are the main autophagy-regulatory pathways in DKD (20) and maintain cellular homeostasis in DKD. mTOR complex 1 (mTORC1) activation is involved in the early stages of DKD (21, 22). Furthermore, specific activation of mTORC1 in podocytes induces DKD-like renal damage, including podocyte effacement, glomerular basement membrane thickening, mesangial expansion, epithelial-mesenchymal transdifferentiation, and proteinuria (23). Increased mTORC1 activity in the proximal tubule in diabetes induces renal fibrosis and renal function decline (24), and targeted mTORC1 inhibition by rapamycin and sodium-glucose cotransporter 2 inhibitors (SGLT2i) is renoprotective in DKD (24–26). In contrast to mTORC1, AMPK is a positive regulator of autophagy. AMPK can regulate autophagy by direct phosphorylation modification, as well as induce autophagy by inhibiting mTORC1. Loss of AMPK aggravates proteinuria in DKD (27). AMPK activation ameliorates apoptosis and fibrosis in DKD (28, 29), and the effect of metformin mitigates renal oxidative stress and fibrosis in DKD is also associated with activating AMPK (30). Notably, AMPK also plays an important role in maintaining mitochondrial homeostasis and optimizing oxidative phosphorylation to maintain energy homeostasis in DKD (31). A recent study found that AMPK plays a central role in the amelioration of kidney injury in diabetes nephropathy (DN) by the vitamin D (VD)-vitamin D receptor (VDR). VD-VDR activates AMPK to regulate autophagy in DN in a calcium-dependent manner (32). SIRT1, an NAD^+^-dependent deacetylase, plays a protective role in kidney disease (31). SIRT1 can directly deacetylate Beclin1 to activate autophagy (33) and regulate the expression of autophagy-related proteins (34), as well as regulate autophagy by serving as a substrate (35, 36). Induced SIRT1-overexpression in podocytes attenuates proteinuria and glomerular injury in DKD (37, 38). Furthermore, SIRT1 mediates communication between proximal tubules and podocytes, contributing to maintaining the nicotinamide mononucleotide concentration around glomeruli, which is essential for preventing podocyte injury and proteinuria in DKD (39, 40).

### 2.2 Mitophagy and lipophagy

Mitophagy, which contributes to mitochondrial quality, is an important selective autophagy mechanism in DKD, as impaired mitochondrial function and abnormal mitochondrial accumulation are involved in DKD onset and progression (41, 42). Mitophagy inhibition aggravates tubulointerstitial inflammation and premature tubular cell aging (43, 44). Similarly, mitophagy has also been implicated in podocyte energy metabolism, inflammation, and apoptosis (45), and promoting mitophagy homeostasis contributes to the amelioration of podocyte injury in DKD (46).

Lipid metabolism disorders and autophagy imbalance are the main characteristics of DKD, both of which are rapidly developing areas of research (47, 48). Ectopic lipid deposition (ELD) accelerates renal resident cell injury and senescence (49). Lipophagy improves ELD and attenuates lipotoxicity-induced kidney injury (50). Furthermore, lipophagy reduces cholesterol influx and ameliorates lipotoxicity-induced podocyte injury and tubular injury in DKD (51, 52).

### 2.3 Autophagy and kidney inflammation

Crosstalk between autophagy and inflammation is widespread and important in many diseases (53), including DKD. Inflammation plays a key role in DKD onset and progression, and autophagy plays multiple roles in the inflammatory response (54). The accumulation of AGEs triggered the inflammatory response in DKD (55), and autophagy as an important regulator ameliorated renal inflammation by promoting the degradation of AGEs (56). Furthermore, autophagy-related gene 5 (ATG5) ablation was found to impair autophagy and enhance NF-κβ activation (10). Notably, lysosomal rupture also leads to inflammasome activation, further aggravating inflammation (57). TFEB, an important transcription factor for autophagy and lysosomal regulation, plays a multifaceted role in regulating macrophage activation and control cytokine/chemokine transcription (58). NLRP3 inflammasome is an important player in the regulation of inflammation. NLRP3 inflammasome activation impairs glomerular autophagy in DKD, and NLRP3 inhibition or deletion is sufficient to restore autophagy in podocytes (59), highlighting the close relationship between inflammation and autophagy in DKD. Thus, targeted elimination of the crosstalk between autophagy and inflammation has a promising therapeutic effect on DKD (35, 60, 61).

### 2.4 Lysosomal dyshomeostasis

Lysosomes play invaluable roles in various types of autophagy and cell death (62), are dynamic regulators of cellular and organismal homeostasis, and are responsible for the degradation of cellular content (63). Lysosomes contribute to cellular metabolism, membrane repair, and immune signal transduction (64) and also communicate extensively with other organelles, including mitochondria (65) and the nucleus (66), by establishing membrane contact sites and functional interactions. Lysosome-related research on DKD is advancing rapidly. The accumulation of AGEs triggers lysosomal membrane permeability and lysosomal dysfunction (67). Conversely, lysosomal biogenesis promotes degradation of AGEs in DKD (55). Restoring lysosomal function to activate autophagy improves podocyte damage in DKD (12), and a similar effect has been observed in renal tubules (55). Interestingly, recent studies have found that tunneling nanotubes (TNT) mediated the exchange of autophagosomes and lysosomes between podocytes to allow healthy podocyte components to replace damaged organelles, and the inhibition of TNT accelerated lysosomal dysfunction and apoptosis in podocytes (68). The inactivation of TFEB, a master transcription factor that drives lysosomal functions, is closely related to lysosomal insufficiency and dysfunction in DKD (69). AGEs inhibit the nuclear translocation and activity of TFEB (70). Pharmacologically targeting TFEB activation ameliorates tubular and podocyte injury, apoptosis, and inflammation in DKD (69–71). Maintaining lysosomal homeostasis may therefore be a potential therapeutic approach for DKD (7, 72).

## 3 Autophagy and renal homeostasis

**Figure 2:**
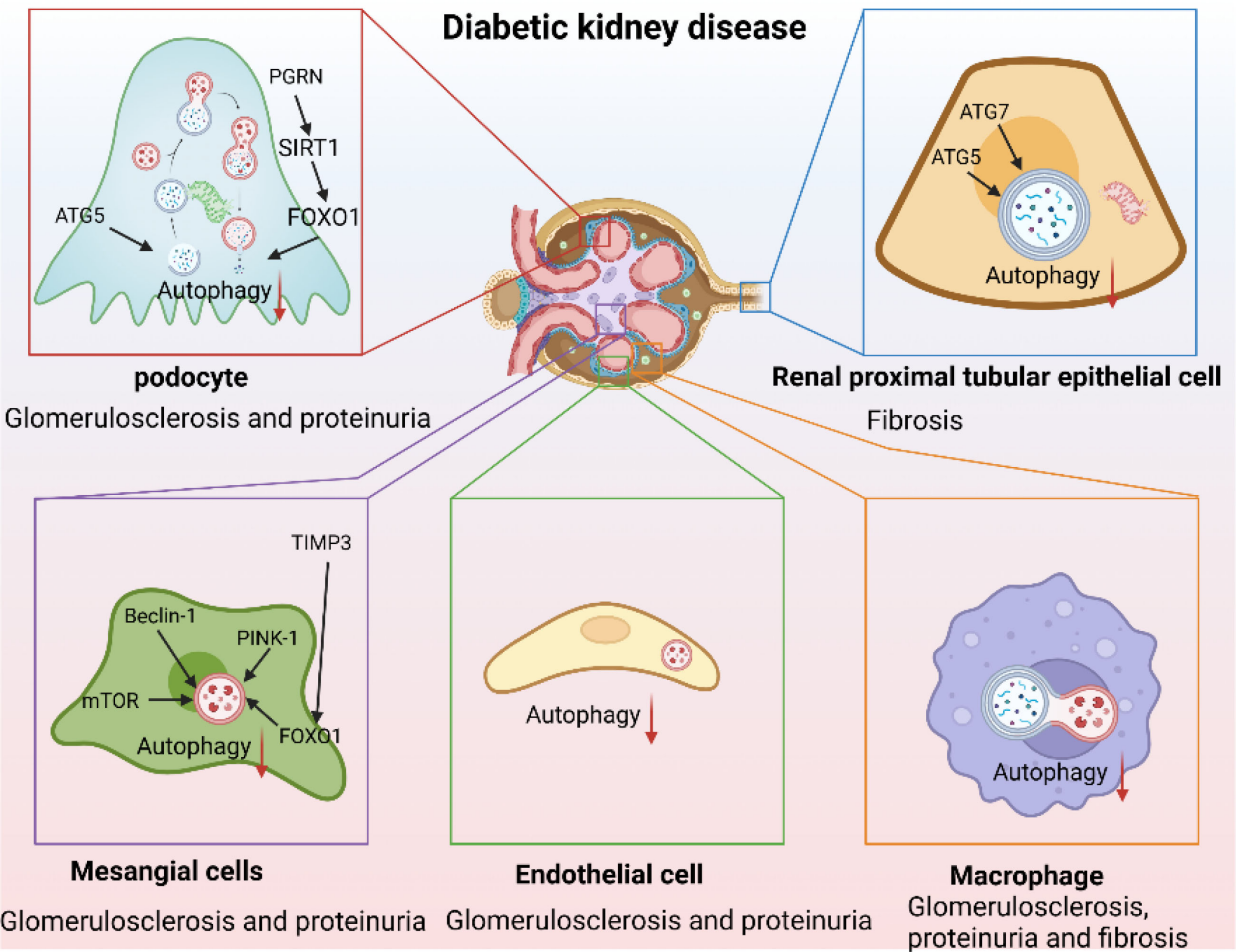
Autophagy as a therapeutic target in DKD. (Autophagy is inhibited in DKD. Autophagy-lysosome dysfunction mediates podocyte injury leading to glomerulosclerosis and massive proteinuria and mediates renal proximal tubular epithelial cells injury aggravating renal fibrosis, and mediates mesangial cell injury and endothelial-to-mesenchymal transition promoting the production of mesangial matrix and aggravating glomerulosclerosis and proteinuria. In addition, inhibition of macrophage autophagy aggravated renal inflammation leading to glomerulosclerosis, proteinuria, and renal fibrosis).

Autophagy functions differently in various cell types. In this section, we review studies on autophagy in four important cell types implicated in DKD: podocytes, tubular epithelial cells, mesangial cells, endothelial cells, and macrophages (**Table 1**, **Figure 2**).

**Table 1:** Role of autophagy dysregulation in major cell types affected in DKD.

Cell types	Experimental models	Major findings	References
Podocyte	/	The physiologic function of podocytes requires the maintenance of high levels of autophagy and is independent of mTOR	(73)
	HFD-Induced Diabetes in Podocyte-Specific Autophagy-Deficient Mice	Autophagy deficiency accelerates podocyte loss and proteinuria	(11)
	STZ-induced DKD	Inhibition of mitophagy accelerates podocyte injury	(46)
	STZ-induced DKD	Autophagy promotes the degradation of cholesterol and ameliorates podocyte injury resulting from lipotoxicity	(51)
	STZ-induced DKD	Loss of autophagy accelerates podocyte injury, disruption of the glomerular filtration barrier and glomerulosclerosis	(74)
Tubular epithelial cell	AGE-induced podocyte injury	Lysosome restoration activates autophagy to ameliorate podocyte injury	(12)
	/	the basal level of autophagy in renal PTECs is very low	(8)
	/	Apoptosis and senescence of tubular cells accelerated by autophagy deficiency	(9)
	STZ-induced DKD	Knockdown of SGLT2 aggravates the impaired autophagy	
		Lysosomes of tubule cells promote degradation of AGEs	(14)
	/	Autophagy mediates tubulobulbar feedback	(75)
Mesangial cell	AGEs induced HBZY-1 cells	AGEs induce autophagy alterations in a time-dependent manner	(76)
	HFD/STZ-induced DKD	Autophagy improves the expansion of glomerular mesangium and the deposition of extracellular matrix	(77)
	high glucose induced SV40 MES 13	Autophagy dysfunction aggravates inflammation and fibrosis	(78)
	AGEs induced HBZY-1 cells	Autophagy clear ROS and repair AGEs induced MCs damage	(79)
	/	Inhibition of autophagy accelerates senescence in MCs	(80, 81)
Glomerular endothelial cells	STZ-induced DKD	ECs specific deletion of Atg5 leads to capillary rarefaction and accelerated DN	(74)
	HFD/STZ-induced DKD	Autophagy improved the proliferation and inhibited apoptosis of GECs	(82)
	db/db mice	Autophagy inhibition promoted endothelial-to-mesenchymal transition	(83)
Macrophage	STZ-induced DKD	Activation of TFEB and recovery of autophagy promote M2 polarization of macrophages	(71)
	HG induced RAW264.7	Autophagy promotes macrophage adhesion and migration in DKD	(84)
	STZ-induced DKD	Mitophagy regulates macrophage phenotype in DKD	(85)
	HG induced RAW264.7	High glucose induced macrophage derived exosomes promote renal tubular epithelial cell autophagy inhibition	(86)
	HG-induced podocyte injury	M2 macrophage derived exosomes activate autophagy to ameliorate podocyte injury	(87)

HG, high glucose; STZ, Streptozocin; HFD, High fat diet; DKD, diabetic kidney disease; AGEs, advanced glycation end products; EndMT, endothelial-to-mesenchymal transition.

### 3.1 Autophagy and podocyte injury

Podocytes are terminally differentiated glomerular epithelial cells that play key roles in maintaining the integrity of the glomerular filtration barrier. Podocyte injury and effacement are the main causes of glomerulosclerosis and massive proteinuria in patients with DKD. The physiological function of podocytes requires high levels of autophagy (73). Podocyte-autophagy inhibition occurs in the early stages of DKD and leads to the progression of DKD and massive proteinuria (11). Loss of autophagy was found to simultaneously accelerate podocyte and endothelial injury, leading to disruption of the glomerular filtration barrier and glomerulosclerosis (74). Mitophagy is also involved in podocyte injury. For example, progranulin (PGRN) deficiency aggravates podocyte injury and proteinuria in DN mice, while elevated PGRN levels maintain podocyte mitochondrial homeostasis by mediating mitochondrial biogenesis and mitophagy via the SIRT1-PGC-1α/FoxO1 pathway (46). Similarly, forkhead-box class O1, a transcription factor, reduces podocyte injury in DKD by regulating mitophagy (88). Recent evidence suggests that autophagy aids in the reduction of ELD and the amelioration of lipotoxicity-mediated podocyte injury in DKD (51). Notably, lysosomes are involved in the processing of endocytosed albumin in podocytes, lysosomal dysfunction may contribute to podocyte injury, albuminuria, and glomerulosclerosis (89). AGE-stimulation leads to decreased lysosomal enzyme activity, TFEB inactivation, and lysosomal membrane permeabilization in podocytes (12). This results in an inhibition of the autophagic flux, resulting in podocyte actin cytoskeletal disorganization and loss of slit membrane integrity. Pharmacological attenuation of autophagy and lysosomal dysfunction with drugs such as rapamycin, reduces proteinuria and ameliorates podocyte injury in DKD (90). These studies highlight the importance of autophagic flux in maintaining podocyte homeostasis.

### 3.2 Autophagy and renal proximal tubular epithelial cells

Although DKD is traditionally characterized by glomerulopathy, many patients with DKD who develop ESRD do not show increased proteinuria (91), illustrating the importance of renal proximal tubular epithelial cell (PTEC) damage for the onset and progression of DKD. Phenotypic changes in PTECs are early manifestations of DKD (92, 93), and the severity of tubulointerstitial lesions strongly correlates with renal outcomes (94). The autophagy-lysosome pathway is important in PTEC, although, unlike in podocytes, the basal level of autophagy in renal PTECs is very low (8). Mice with proximal tubule-specific ATG5 or ATG7 deletion exhibit exacerbated renal function impairment and premature renal senescence (8, 9). Furthermore, deleting sodium-glucose cotransporter 2 (SGLT2), a master regulator of renal tubular glucose reabsorption, reduces renal p62/SQSTM1 accumulation, suggesting that glucose uptake may contribute to autophagy inhibition in PTECs (95). AGEs, which are elevated by long-term HG levels, are degraded by endocytosis in PTEC lysosomes and gradually accumulates with the PTEC autophagy impairment (14). The AGE-overload, in turn, disrupts lysosomal function and autophagic flux, aggravating PTEC injury (55, 67). Impaired mitophagy plays a fundamental role in DKD pathogenesis, and mitophagy deficiency in PTECs leads to tubular cell injury and accelerated senescence (43). MitoQ, a mitochondria-targeted anti-oxidant, attenuates tubular injury and improves renal function by enhancing mitophagy (96). It is important to note that tubular injury often coincides with glomerular injury in DKD, forming tubuloglomerular feedback (TGF) (97), and autophagy plays an indispensable role in TGF through Megalin and SIRT1 (75, 98). Collectively, these studies indicate that PTEC autophagy plays an important renoprotective role in DKD.

### 3.3 Autophagy and mesangial cells

Mesangial cells (MCs) play an important role in maintaining the structural integrity of the glomerular microvascular bed and mesangial matrix homeostasis (99) by eliciting multiple biological responses to injury, including matrix remodeling and crosstalk with neighboring cells (100). MC hypertrophy, basement membrane thickening, and mesangial matrix expansion induced by HG levels are the earliest pathological features of DKD (101). However, the role of autophagy in MCs in DKD remains unclear. AGE-stimulation of MCs leads to time-dependent changes in LC3II and p62 expression (76). Similarly, HG-stimulation of MCs suppresses autophagy-related protein levels, including mTOR, Beclin1, P62, PINK1, and Parkin. Moreover, inhibition of autophagy in MCs is also found to accelerate AGE-induced senescence in MCs (80, 81) and aggravate renal inflammation and fibrosis in DKD (78). A loss of the tissue inhibitor of metalloproteinase 3 (TIMP3) aggravates basement membrane thickening, mesangial expansion, proteinuria, and interstitial fibrosis in DKD, resulting in decreased expression of FoxO1 and autophagy-related genes, while re-expression of TIMP3 in MCs attenuates these effects (102). Activation of MC autophagy can repair AGEs-induced MC damage by clearing reactive oxygen species (ROS), which are important mediators of AGE-induced MC-apoptosis (79), and improve glomerular mesangial expansion and extracellular matrix deposition, thereby improving DKD (77). Although these studies provide important evidence for the protective role of autophagy in MCs, the potential therapeutic value of autophagy in MCs in DKD requires further research.

### 3.4 Autophagy and glomerular endothelial cells

Glomerular endothelial cells (GECs) injury plays a key role in the early occurrence and development of DKD (103). High glucose, ROS accumulation, and autophagy inhibition mediate the loss of glycocalyx and GECs dysfunction, leading to endothelial permeability and apoptosis, thereby driving albuminuria and early renal injury (104, 105). Importantly, injured GECs accelerates renal progression by forming crosstalk with adjacent glomerular cells (82, 106). Conditional knockout of Atg5 of GECs showed capillary loops thickening and accumulation of ROS, which eventually developed significant glomerulosclerosis (107). In addition, knockout of Atg5 GECs leads to capillary rarefaction and endothelial-to-mesenchymal transition (EndMT) and accelerating the fibrosis and progression of DN (74, 83, 108). Interestingly, the injured of GECs promoted podocyte dysfunction (109). In turn, autophagy of podocytes plays a renoprotective role against DKD related structural endothelial injury (110). Activating the AMPK pathway was proved to improve the renal injury of DKD by improving autophagy of GECs (29, 111), suggesting the important role of autophagy of GECs in improving DKD.

### 3.5 Macrophage autophagy in DKD

DKD is a chronic inflammatory disease characterized by massive inflammatory cell infiltration and overexpression of proinflammatory factors. Increased macrophage infiltration is observed in the kidneys of DN mice and patients with DKD. Macrophage infiltration and phenotypic changes are significantly associated with proteinuria and fibrosis in DKD (112). Furthermore, communication between macrophages and renal resident cells, such as podocytes and PTECs, may influence DKD progression (113, 114). Emerging evidence suggests that macrophage autophagy plays a crucial role in macrophage polarization, chronic inflammation, and organ fibrosis (115). In DKD, HG-stimulation results in macrophage-derived exosomes targeting and inhibiting PTEC autophagy (86), and M2 macrophage-derived exosomes activate autophagy to ameliorate podocyte injury in DKD (87). Conversely, autophagy can regulate changes in the macrophage phenotype (85), and autophagy inhibition enhances macrophage adhesion and migration (84). Furthermore, studies have reported that TFEB activation promotes macrophage polarization toward the M2 type, suppresses inflammation, and improves kidney injury in DKD (71). Targeting autophagy regulation in macrophages has been well-studied in many diseases (116, 117). Macrophage autophagy may therefore be a promising therapeutic target for DKD.

## 4 Polyphenols used to regulate autophagy in DKD

**Figure 3:**
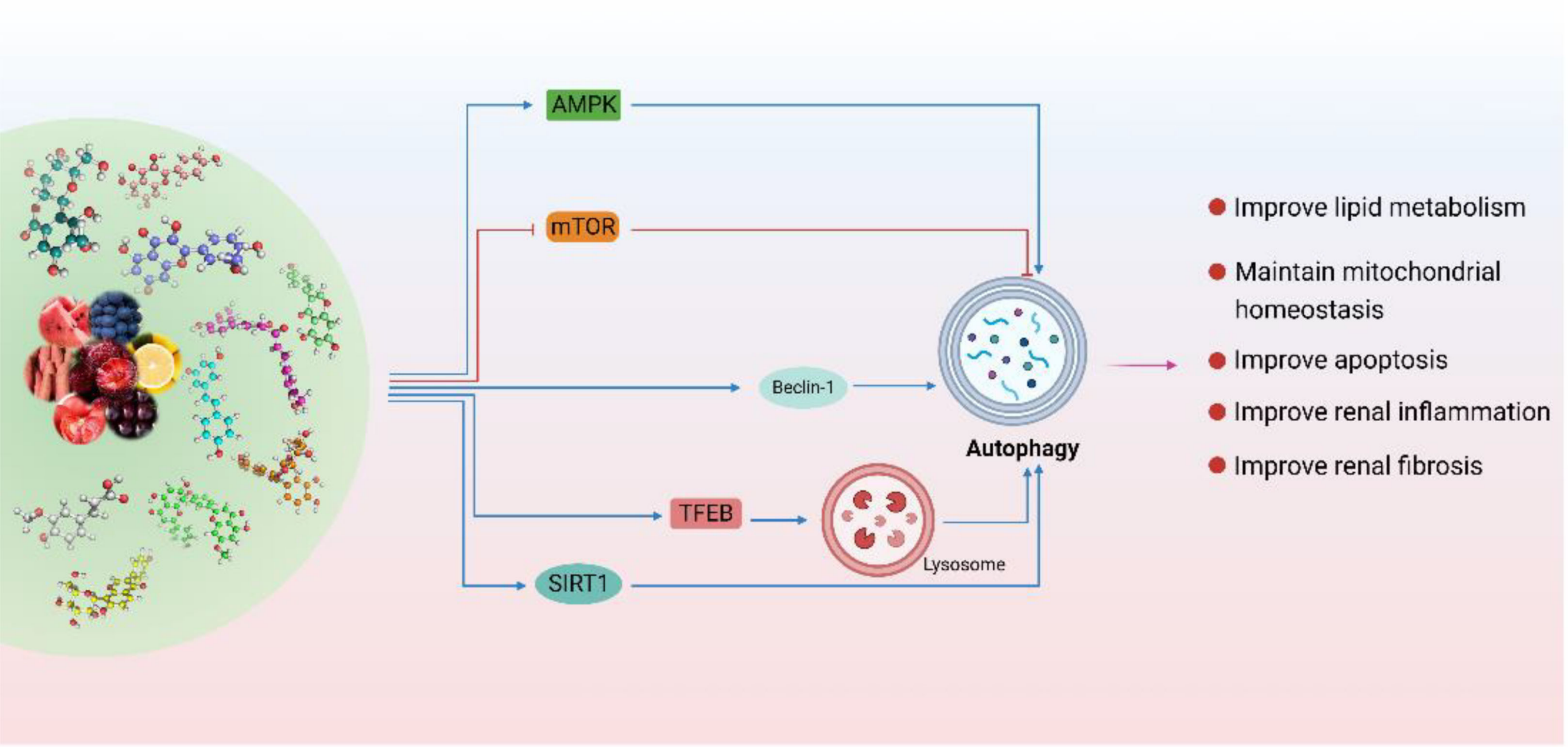
Polyphenols are used to regulate autophagy in DKD. (Natural polyphenols are potential autophagy regulators. Polyphenols are mainly through the SIRT1 pathway, mTOR pathway, and AMPK pathway is involved in the regulation of various links of autophagy. In addition, polyphenols can also improve the autophagy-lysosome pathway by activating TFEB and promoting its nuclear translocation. In conclusion, polyphenols play an important role in improving DKD by regulating autophagy-lysosome pathway to improve renal inflammation, lipid metabolism, mitochondrial homeostasis, apoptosis, and fibrosis.

Dietary polyphenols are a widespread class of secondary plant metabolites. The potential of polyphenols to restore SIRT1 and NAD^+^ metabolism in kidney diseases has received significant attention (118). In addition, epigenetic regulation of autophagy is an important mechanism for maintaining homeostasis. Natural polyphenols can reverse epigenetic alterations of autophagy and delay the progression of DKD (119). Resveratrol was found to regulate SIRT1 and DNA-methyltransferase (DNMT) and exhibited potential regulatory capacity on DKD (120, 121). Similarly, quercetin has also been found to regulate the expression of multiple chromatin modifiers (including DNMTs, histone deacetylases, histone acetyltransferases, and histone methyltransferases) (122). In this section, we review natural polyphenols as autophagy regulators, including the regulation of mitophagy and promoting TFEB-nuclear transfer. The reviewed compounds and their specific effects on autophagy regulation are summarized in **Table 2** (**Figure 3**).

**Table 2:** Summary of natural polyphenols targeting autophagy to improve DKD.

Compounds	Types of study	Source	Model	Molecular targets	Pathway	Modulation on autophagy	Effects	References
Resveratrol	*In vitro*, *In vivo*	Wine, berries, and peanuts	STZ induced DKD; db/db mice;	LC3-II/LC3-I↑, p-AMPKα/AMPKα↑, p-ULK1↑, SIRT1↑, Atg7↑, Atg5↑, LC3↑	AMPKα/mTOR; SIRT1	Improve autophagy; Restore lysosome function	Improve insulin resistance, lipid metabolism and renal function	(12, 123–126)
Curcumin	*In vitro*, *In vivo*	*turmeric*	STZ-induced DKD	LC3II/LC3I↑, p62↓, p-mTOR↓, UVRAG↑, p-Akt↓, P13K↓, Atg5↑, Beclin-1↑	PI3k/Akt/mTOR; Beclin1/UVRAG/Bcl2	Improve autophagy	Improve podocytes EMT and apoptosis	(127–129)
Puerarin	*In vitro*, *In vivo*	*radix puerariae*	STZ-induced DKD	LC3-II↑, p62↓, Beclin-1↑, Atg5↑, LKB1↓	HMOX1/SIRT1; PERK/eIF2α/ATF4	Improve autophagy	Protect podocytes from damage induced by diabetes	(130, 131)
Kaempferol	*In vitro*, *In vivo*	tea leaves, broccoli, hazelnuts, propolis, grapefruit and other green plants	db/db mice	LC3II↑, Beclin-1↑, Atg7↑, Atg 5↑, p62/SQSTM1↓, p-AMPK↑, p-mTOR↓	AMPK/mTOR	Promotes autophagy;	Amelioration of podocyte injury and renal cell apoptosis	(132, 133)
Cyanidin-3-O-glucoside	*In vitro*	anthocyanins	HG- mouse podocytes(MPC5)	LC3-II/LC3-I↑, Beclin-1↑, p62↓, p-AMPK/AMPK↑, p-mTOR/mTOR↓, SIRT1↑	SIRT1/AMPK	Improve autophagy and apoptosis	Improve podocytes EMT and apoptosis	(134)
Ferulic acid	*In vitro*, *In vivo*	*Ferula asafoetida L., Anthemis nobilis L. and Equisetum hyemale L.*	HFD/STZ-induced DKD	LC3-II/LC3-I↑, p62↓	/	Improve autophagy	Improve renal injury	(135)
isorhamnetin	*In vitro*, *In vivo*	the fruits of *Hippophae rhamnoides L.* and the leaves of *Ginkgo biloba L.*	HFD/STZ-induced DKD	FYCO1↑, ULK-1↑, TECPR↑, WIPI↑	/	Improve autophagy	Improve fasting blood glucose, lipid metabolism and renal function	(136)
phenolics from Physalis Peruviana fruits	*In vitro*, *In vivo*	*Physalis peruviana* fruits	STZ-induced DKD	LC3-II↑, AMPK↑, mTOR↓	AMPK/mTOR	Improve autophagy and apoptosis	Improve renal injury	(137)
Wogonin	*In vitro*, *In vivo*	the root of Scutellaria baicalensis Georgi	STZ-induced DKD	ATG7↑, LC3-II/LC3-I↑, Beclin-1↑, p62↓	PI3K/Akt/NF-κB	Regulation of crosstalk between autophagy and apoptosis	Attenuate podocyte injury; regulate the crosstalk between autophagy and apoptosis to reduce glomerulopathy and podocyte damage	(138, 139)
Dihydromyricetin	*In vitro*, *In vivo*	*Ampelopsis Michx*	STZ-induced DKD	LC3-II/LC3-I↑, Beclin-1↑, p62↓,	PI3K/AKT/mTOR	Improve autophagy	Improve renal interstitial fibrosis	(140)
Genistein	*In vitro*	soy	HG-induced podocyte	LC3-II↑, p62↓, p-mTOR↓	mTOR	Improve autophagy	Improve podocyte damage	(141)
Salvianolic Acid A	*In vitro*, *In vivo*	dried root and rhizome of *Salvia miltiorrhiza Bunge*	HFD/STZ-induced DKD	SIRT1↑, ATG5↑, ATG7↑, ATG12↑, LC3-II↑, Beclin-1↑, p62↓, Bnip3↑	Sirt1-Foxo3a-Bnip3	Ameliorate the impaired autophagy	Restored glomerular endothelial function and alleviated renal structural deterioration	(142)
Bergenin	*In vitro*, *In vivo*	*Bergenia crassifolia, Ficus racemosa, Mallotus japonicus, M. philippinensis, etc*	HFD/STZ-induced DKD	p-mTOR↓	mTOR/β-TrcP/Nrf2	/	Inhibit the generation of extracellular matrix in glomerular mesangial cells	(143)
Ginkgetin	*In vitro*	*Ginkgo biloba* leaves	HG- induced rat glomerular mesangial cells	LC3-II/LC3-I↑, p62↓, p-MPK↑, p-mTOR↓	AMPk/mTOR		Mesangial cell oxidative stress injury, inflammation, and extracellular matrix deposition	(144)
Chrysin	*In vitro*, *In vivo*	Edible plants such as passion flowers, mushrooms, honey, and bee propolis	*db/db* mice	Atg3↓, Atg7↓, Beclin-1↓, LC3-I/LC3-II↓, mTOR↓, p-mTOR↓	mTOR	Inhibition of Autophagic	Inhibit mesangial actin assembly and cell migration	(133, 145)
Mangiferin	*In vitro*, *In vivo*	*Salacia oblonga*	HFD/STZ-induced DKD	P62↓, LC3-II/LC3-I↑, p-mTOR↓, p-AMPK↑, p-ULK1↑	AMPK-mTOR-ULK1	Ameliorate the impaired autophagy	Improve podocyte damage	(146)
Fisetin	*In vitro*, *In vivo*	Various fruits and vegetables	*eNOS^−/−^ mice*	P62↓, LC3-II/LC3-I↑	CDKN1B/P70S6K	Restored autophagy	Improve podocyte damage	(147)
Isorhapontigenin	*In vitro*, *In vivo*	*Gnetum cleistostachyum*	STZ-induced DKD	Beclin-1↑, P62↓, Atg5↑, p-AMPK↑	AMPK/Nrf2	Activate autophagy and reduce oxidative stress	Improve podocyte and endothelial cell damage	(111)

HG, high glucose; STZ, Streptozocin; HFD, High fat diet; DKD, diabetic kidney disease; EMT, epithelial-mesenchymal transition.

↑increase; ↓decrease.

### 4.1 Resveratrol

Resveratrol (RSV), a potent, natural SIRT1-agonist, reduces oxidative stress and AGE production, inhibits endoplasmic reticulum (ER) stress, and ameliorates lipotoxicity and inflammation, thereby effectively protecting renal function from DKD without significant side effects, and is widely recommended as a dietary supplement for DKD treatment (148). RSV is also an effective autophagy regulator in DKD, promoting autophagy by activating SIRT1, thereby ameliorating DKD (123). RSV enhance autophagy to improve insulin resistance, lipid metabolism, and renal function in DKD (124), and protects against HG-induced podocyte apoptosis, whereas inhibition of autophagy reverses this therapeutic effect (125). Further research found that RSV can further improve autophagy and apoptosis in podocytes by up-regulating miR-18a-5p (126) and down-regulating miR-383-5p (125). Furthermore, RSV, in combination with vitamin E, improves the lysosome-dependent autophagy pathway, thereby ameliorating AGE-mediated podocyte actin cytoskeleton damage (12).

### 4.2 Curcumin

Curcumin, a bioactive polyphenolic compound found in *turmeric*, exhibits anti-inflammatory, anti-oxidant, autophagy-enhancing, anti-apoptotic, and anti-fibrotic properties in DKD (149). Moderate doses of curcumin induce autophagy, whereas high doses induce lysosomal membrane permeabilization, leading to cell death (150). Curcumin has been described as a pharmacological inhibitor of the mTOR-signaling pathway in many diseases (151, 152). Curcumin also activates TFEB, thereby enhancing autophagy and lysosomal activity (153, 154). In DKD, curcumin suppresses p-mTOR levels, thereby promoting autophagy and alleviating podocyte epithelial-to-mesenchymal transition (127), and ameliorates podocyte apoptosis via Beclin1/UVRAG/Bcl2 pathway (128). In addition, autophagy has also been shown to reduce AGEs induced apoptosis in tubule cells (129).

### 4.3 Puerarin

Puerarin, an isoflavone extracted from *Pueraria lobata*, is widely used in traditional Chinese medicine. Clinical and basic studies have shown that puerarin exerts renoprotective effects (155, 156) and that autophagy is the primary mechanism by which puerarin alleviates DKD. Puerarin activates autophagy to promote podocyte functional protein expression under ER stress in DKD (130); acts as a SIRT1-agonist, ameliorating podocyte injury, and proteinuria by activating SIRT1 (40); and promotes heme oxygenase 1 and SIRT1 expression and decreases liver kinase B1 acetylation, thereby activating autophagy to protect podocytes (131).

### 4.4 Kaempferol

Kaempferol, a natural flavanol common in traditional medicines, fruits, and vegetables, is a histone deacetylase (HDAC) inhibitor (157) and a promising autophagy modulator exhibiting therapeutic effects in many diseases (158–160). Kaempferol can not only promote the expression of autophagy-related genes (161) but also promote autophagy in macrophages and inhibit NLRP3 inflammasome activation (162). Recent machine-learning screening has also identified kaempferol as a potent mitophagy inducer for treating Alzheimer’s disease (163). Kaempferol can regulate the AMPK/mTOR pathway to promote autophagy, thereby alleviating mesangial matrix expansion, glomerular basement membrane thickening, and podocyte loss or fusion in DKD (132). Further studies have found that kaempferol also ameliorates ROS generation and mitochondrial damage by AGE accumulation in mesangial cells through autophagy (133).

### 4.5 Other polyphenols

Ferulic acid, a phenolic acid present in the seeds and leaves of most plants, has shown satisfactory effects in the treatment of DKD (164). Ferulic acid was shown to improve renal injury in patients with DKD by enhancing autophagy and inhibiting excessive inflammation (135). Similarly, isorhamnetin, found in *Acanthus nigricans* fruits and *Ginkgo biloba* leaves, ameliorates renal injury in DN by enhancing autophagy and inhibiting excessive inflammation (136). Cyanidin-3-O-glucoside activates SIRT1 and AMPK to ease HG-induced autophagy inhibition, thereby attenuating podocyte dysfunction and epithelial-mesenchymal transition (134). Phenolics from *Physalis peruviana* fruits activate the AMPK/mTOR pathway, enhancing autophagy and ameliorating apoptosis and kidney injury in DKD (137). Similarly, ginkgetin reduces HG-induced oxidative stress damage, inflammation, and extracellular matrix deposition in mesangial cells via AMPK/mTOR pathway-mediated autophagy (144). Genistein has also been found to inactivate the mTOR pathway, maintain autophagy-related protein levels, and inhibit HG-induced podocyte injury (141). Bergenin, a plant polyphenol derived from the cortex of *Mallotus japonicus (L.f.) Müll.Arg.*, and defined as a PPARγ-agonist (165), inhibits oxidative stress and reduces extracellular matrix production in DKD mesangial cells by inhibiting mTOR phosphorylation (143). Wogonin targets phosphoinositide 3-kinase (PI3K) to regulate autophagy and inflammation and attenuates tubulointerstitial fibrosis and tubular cell injury in DN (138). In addition, wogonin has also been found to regulate the crosstalk between autophagy and apoptosis to reduce glomerulopathy and podocyte damage (139). Similarly, dihydromyricetin enhances autophagy and attenuates renal interstitial fibrosis in DN via the PI3K/AKT/mTOR pathway (140). Salvianolic acid A restores the actin cytoskeleton rearrangement of glomerular endothelial cells in DN by modulating autophagy and inflammation via the AGE-RAGE-Nox4 axis, thereby ameliorating early renal injury in DN (142). Chrysin inhibits AGEs-induced activation of the mTOR pathway and promotes autophagy to inhibit mesangial cell proliferation, α-smooth muscle actin production, and adhesion in DN (145). Mangiferin, a xanthonoid from *Salacia oblonga*, can promote the phosphorylation of AMPK and ULK1, inhibit the phosphorylation of mTOR, increase the number of autophagosomes and thereby ameliorate podocyte injury and proteinuria in DN (146). Fisetin has also been found to reduce podocyte injury in DN by restoring autophagy and inhibiting the NLRP3 inflammasome (147). Isorhapontigenin attenuates HG-induced oxidative stress and activates autophagy by stimulating AMPK/Nrf2 pathway, thereby improving the EndMT and podocyte injury of DKD (111).

## 5 Autophagy as a therapeutic target in DKD

Accumulating evidence indicates that autophagy plays a critical role in both early and late stages of DKD. Pharmacological activation of autophagy has shown invaluable advantages in DKD. Rapamycin can reduce streptozocin (STZ)-induced renal injury by promoting podocyte autophagy and inhibiting apoptosis (166). Sirolimus has also been found to block mTOR to reduce fibrosis and mesangial matrix accumulation in STZ-induced DKD (167). In addition, several effective drugs against DKD, such as SGLT2 and metformin, also enhance autophagy. It has been reported that the amelioration of tubulointerstitial fibrosis by SGLT2i in Akita diabetic mice is entirely dependent on mTORC1, and deletion of mTORC1 reverses the renoprotective effects of SGLT2i (24, 25). Similarly, Metformin has also been found to enhance autophagy of mesangial cell via SIRT1 and AMPK pathways to effectively ameliorate glycolipid metabolic disorders, inflammation, MC proliferation, and extracellular matrix expression in DKD (168, 169). The beneficial effects of some non-pharmacological DKD therapies, such as diet and exercise, are also associated with autophagy regulation. Dietary modification was shown to activate SIRT1 and AMPK and inhibit mTOR to regulate autophagy, thereby playing a crucial role in improving DKD (170, 171). A cross-sectional study involving 229 participants found that exercise can improve proteinuria and plasma lipids in patients with diabetes (172), and this renoprotective effect of exercise was found to be associated with activation of AMPK and inhibition of mTORC1 in Wistar fatty (fa/fa) rats (173).

## 6 Therapeutic potential of polyphenols targeted autophagy for DKD

Natural polyphenols as autophagy regulators have shown promise in the treatment of DKD. Many clinical studies have demonstrated the protective effects of dietary polyphenols against DKD. For example, resveratrol was shown to assist angiotensin receptor blockers in reducing proteinuria for DKD patients in a randomized double-blind placebo-controlled clinical trial with 60 participants (174). Similarly, curcumin has also been shown to improve urinary microalbumin excretion and inflammation in a randomized, double-blind, and placebo-controlled study that enrolled 40 patients with overt type 2 DKD (175). Other polyphenols have also been shown to similarly ameliorate podocyte injury in clinical studies, such as green tea polyphenols (176). Based on the preclinical evidence that polyphenols improve autophagy presented above, it is plausible to conclude that polyphenols show therapeutic potential to improve DKD by modulating autophagy. However, there are some challenges in replicating the beneficial effects of polyphenols in clinical settings, such as polyphenol absorption and bioavailability, which may be addressed with new methods and technologies, such as nanotechnology (177).

## 7 Conclusions and future perspectives

Although considerable progress has been made in DKD treatment in recent years, delaying DKD progression remains a global challenge. Autophagy plays a crucial role in DKD onset and progression, with impaired autophagy and lysosomal function aggravating renal resident cell injury and apoptosis, as well as inducing macrophage phenotype changes, resulting in the development of proteinuria and fibrosis in DKD. A growing body of evidence suggests that polyphenol-rich natural products may assist with DKD while causing no serious side effects. The protective effects of polyphenols in DKD involve multiple mechanisms of action, including modulation of inflammation, oxidative stress, autophagy, and mitochondrial quality control. Although polyphenols are generally considered safe, oxidative stress caused by large amounts of polyphenols can have deleterious effects. Additional studies are therefore needed to determine the optimal polyphenol dosage, and extensive human clinical trials are required to evaluate potential side effects.

## Author contributions

TL, QJ, PL, and YZ designed the study. FM and YW collected the data. TL, LY, and HM analyzed the data and drafted the manuscript. All authors contributed to the article and approved the final version of the manuscript.
